# Severe Wound Infection with *Photobacterium damselae* ssp. *damselae* and *Vibrio harveyi*, following a Laceration Injury in Marine Environment: A Case Report and Review of the Literature

**DOI:** 10.1155/2013/610632

**Published:** 2013-09-19

**Authors:** Jörg Hundenborn, Steffi Thurig, Mechthild Kommerell, Heike Haag, Oliver Nolte

**Affiliations:** ^1^Gemeinschaftspraxis für Orthopädie, Chirurgie, Unfallchirurgie Sportmedizin, Manuelle Medizin/Chiropraktik, Theodor Heuss Straße 1, 78467 Konstanz, Germany; ^2^Laboratory of Dr. Brunner, Mainaustraße 48 a/b, 78464 Konstanz, Germany; ^3^Department of Medical Microbiology and Molecular Microbiology, Laboratory of Dr. Brunner, Mainaustraße 48 a/b, 78464 Konstanz, Germany

## Abstract

Marine microorganisms are uncommon etiologies of skin and skin structure infections, that is, wound infections. We report a case of severe wound infection, caused by the marine *Photobacterium damselae* (Vibrionaceae), in a 64-year-old male patient, returning from Australia. The isolate tested positive for pPHDD1, a plasmid conferring high-level virulence. Furthermore, the wound was coinfected with *Vibrio harveyi*, a halophile bacterium, which has never been reported from human infections before. Identification was achieved by use of Matrix-Assisted Laser Desorption-Ionization Time of Flight Mass Spectrometry (MALDI-TOF) and confirmed by 16S rDNA sequencing. Data retrieval from bibliography was complicated since *P. damselae* has been renamed often with a number of synonyms present in the literature: *Photobacterium damsela*, *Vibrio damselae*, *Vibrio damsela*, *Pasteurella damselae*, and *Listonella damsela*. With all synonyms used as query terms, a literature search provided less than 20 cases published worldwide. A majority of those cases presenting as severe wound infection are even fatal following progression into necrotizing fasciitis. Management with daily wound dressing and antibiotic therapy (ofloxacin empirically, followed by doxycycline after availability of microbiology) led in the reported case to a favorable outcome, which seems to be, however, the exception based on a review of the available literature.

## 1. Introduction

Global traveling contributes to rapid dispersal of pathogens from regions, where they are endemic to countries, where they have never been seen before. Recent developments in medical microbiology, in particular the Matrix-Assisted Laser Desorption-Ionization Time of Flight Mass Spectrometry (MALDI-TOF) technology [1], enable fast and accurate identifications of bacteria, even of taxa, which are rarely seen or not to be identified by standard identification methods. As a consequence, microbiologists are sometimes facing surprising findings, challenging the microbiology mind on how to interpret these. In the current case report, we present such an unexpected finding, which—upon discussion with the attending surgeon—turned out to be a zoonosis of uncommon etiology, caused by marine bacteria of the family *Vibrionaceae*.

Despite their relative infrequency, zoonotic infections by members of the Genus *Vibrio* are well known. Between 1981 and 1993 a total of 168 cases of wound infections (85 of these required hospitalization) were reported from Florida. *Vibrio parahaemolyticus* (33.3%), *Vibrio vulnificus* (28.0%), and *Vibrio alginolyticus* (22.0%) were the most frequently isolated species. Out of the hospitalized patients, 9.4% had a fatal outcome. Other *Vibrio* species, isolated from zoonotic wound infections, were *Vibrio cholerae* (O1 and non-O1 isolates), *Vibrio hollisae* (in the meantime renamed to *Grimontia hollisae*), *Vibrio fluvialis*, *Vibrio mimicus*, *Vibrio damsela*, and *Vibrio metschnikovii*. Further Gram-negative marine bacteria such as *Aeromonas hydrophila* may cause wound infections as well [2, 3]. Infections due to the two most frequent bacteria, *V. parahaemolyticus* and *V. vulnificus*, have recently been identified as an increasing and important clinical problem in Europe [4]. Physicians must be aware that in patients presenting with wound infections and reporting contact to seawater and fish or exposure to seafood, the above-mentioned *Vibrio* species may be the cause of infection. In such cases, early empiric antibiotic treatment is required [3], and bacteriology is imperative. In order to guarantee accurate diagnostic service, it is mandatory to include as much information as possible into the diagnostic request to enable the microbiologist to interpret the findings of bacteriology.

## 2. Case Description

The microbiology unit of a routine laboratory received a wound swab for bacteriology examination. The swab was taken prior to antimicrobial treatment from the infected wound of a 64-year-old male patient. Wound infection at the lower leg was recorded on the documents accompanying the swab, and bacteriology including susceptibility testing was requested. Two highly unusual microorganisms, *Photobacterium damselae* and *Vibrio harveyi*, were isolated. The microbiologists' first suspicion of the patient being a fisherman at the nearby Lake of Constance became unlikely upon identification of *V. harveyi*, since this microorganism is a halophilic inhabitant of seawaters. In fact, the 64-year-old male, athletic, and sportive patient without known underlying conditions had a travel history. He presented with an infected wound following return of a vacation in Western Australia. There, he had been active in aquatics and had done a boat tour in the Indian Ocean near the estuary of Murchison River. He got a laceration injury at the forefront of his right tibia, after falling off a catamaran and having had contact with the sharp-edged keel. The patient consulted a local hospital where the wound was closed with a suture. No antimicrobials were prescribed, and he was discharged from ambulatory care. However, the wound developed signs of inflammation, and the patient presented at a local tertiary hospital upon his return to Germany one week later. Topical ointment and change of bandage were performed. Since the inflammation progressed, the suture was removed and the wound was opened for drainage. Following renewal of the ointment the patient was sent to the surgeon office of one of the authors for continuation of wound management. Here, a wound of approximately 30 × 20 mm in size with a reddish margin of about 60–70 mm was dressed by daily ambulant care. The wound presented at this first visit on day 8 after the injury with necrotic areas and a superficial smear. However, the patient did not show any systemic symptoms like fever, chills, and so forth. Therefore, surgical wound debridement was done, and ethacridine ointment (Rivanol) was applied. Following swabbing for microbiological investigation, empiric antibiotic treatment with ofloxacin (Tarivid), 200 mg twice daily, was initiated. 

## 3. Bacteriology

**Figure 1:**
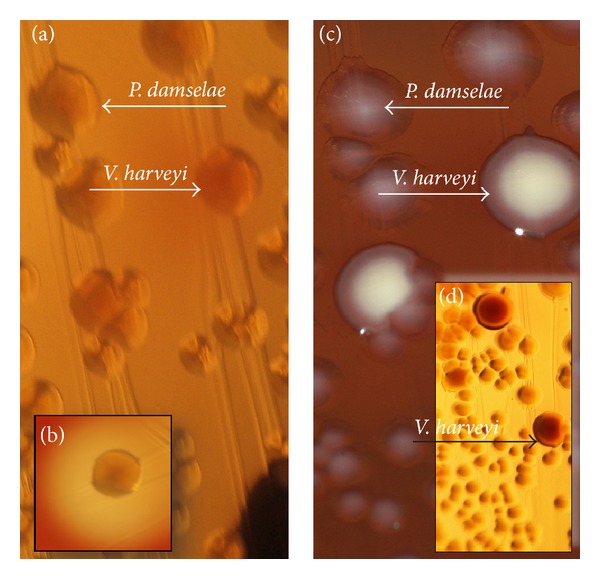
Growth of *P. damselae* and *V. harveyi* on Columbia agar, (a) following 24 and (c) following approximately 48 hrs of incubation, as observed from the original inoculation. The colony marked with *V. harveyi* in (c) has a diameter of 2-3 mm. Note that (a), (b), and (d) were taken with flash against transmitting sunlight while (c) was taken with flashlight from above only. The section in (a) shows the growth after 24 hrs at 35 ± 1°C, and (b) is taken at the same time, but illustrating the hemolysis of *P. damselae*. (c) was taken following additional 24 hrs of incubation, however, at room temperature. While the images (a) and (c) show almost identical sections of the agar plates, they are not superimposable due to variations in the position. (d) illustrates the differences in color, seen especially after approximately 48 hrs, when *V. harveyi* was allowed to grow at a more ambient temperature (i.e., room temperature).

Upon receipt of the swab, culture was initiated following standard procedures. Columbia, MacConkey, and Schaedler agar (bioMérieux, Marcy l'Etoile, France) were inoculated automatically by use of WASP instrument (Copan, Italy). At the first visual inspection of the aerobically (5% CO_2_ atmosphere, 35 ± 1°C) incubated Columbia agar plate after 18–20 hrs, moderate growth of medium-sized, flat, light-grey colonies with extensive *β*-hemolysis was observed. Identification was done, and the plates were left aside for further 24 hrs. With the second visual inspection, few additional larger colonies became apparent, which were not distinguished with the previous reading of the plates (Figure 1). These colonies impressed, in transmitting light, with a red-colored center.

The hemolytic bacteria were identified by use of MALDI-Biotyper MALDI-TOF instrument (Bruker Daltonics, Bremen, Germany), as being *P. damselae*, while—on the next day—the nonhemolytic large colonies were classified as being *V. harveyi* (MALDI-TOF scoring shown in Table 1). While *P. damselae* was found to be catalase negative, with weak reaction against oxidase reagent, *V. harveyi* was positive in both biochemical tests. Both bacteria impressed in the Gram stain as short, partly coccoid but rod-shaped microorganisms.

## 4. Molecular Characterization

In case of rarely encountered species, 16S rDNA sequencing is applied in our laboratory within a course of extended validation of the comparatively new technique of mass spectrometry in routine bacteriology. DNA was therefore extracted from single colonies and the 5′ part of the 16S rDNA amplified as previously described [5]. Primer 27f was used for sequencing, which was done at a commercial sequencing unit in Constance (gatc Biotech, Constance, Germany). Sequences were checked carefully and edited where necessary. Each sequence was BLASTed against the NCBI database [6], with the online tool SepsiTest BLAST [7] and with the “Lightened Version Bio Informatic Bacteria Identification” tool (LeBIBI) [8, 9]. The sequence matched 100% (length of sequence 876 nucleotides) with the GenBank deposited sequence of *P. damselae*, isolate Mm041 [gi*|*334352811] and 99.9% (873 of 874 nucleotides) with that of *P. damselae* ssp. *damselae* in the SepsiTest BLAST database. 

Identification of the second micro-organism was ambiguous in the SepsiTest BLAST database since two hits with identical scores of 99.9% were found, *V. harveyi* and *Vibrio rotiferanus*, while NCBI BLAST provided with 99.9% identity to published sequences *V. harveyi* and *Vibrio communis*. 

Based on the combined results of MALDI-TOF and 16S rDNA analysis, we reported *P. damselae* and *V. harveyi* as final identification. 

In order to examine as to whether the *P. damselae* isolate was of the highly virulent genotype, we subsequently used primer F-P.dam-3 [10] and our newly designed primer R-P.dam-5 (5′ ATCGAACAGTATGCTCTAGGCT 3′) to successfully amplify a 366 bp subgenic fragment of the plasmid located *dly* gene. Sequencing of the amplicon in both directions showed a 100% match with the only available Genbank sequences gi*|*311872675 (*Photobacterium damselae* subsp. *damselae* virulence plasmid pPHDD1, complete sequence, isolate RM71) and gi*|*295427 (*Vibrio damsela* phospholipase D (dly) gene, complete cds).

## 5. Susceptibility Testing

The presence of *β*-lactamase genes in members of the family *Vibrionaceae* has been reported recently [11]. The *P. damselae* isolate was tested on mueller-hinton agar, and the inhibition zones were automatically recorded using the SirScan instrument (i2a, Pérols Cedex, France). Since the genus *Photobacterium* is a member of the family *Vibrionaceae*, interpretation of the inhibition zones (Table 2) was done using the CLSI guidelines for *Vibrio* ssp. (excluding* Vibrio cholerae*), which are implemented in the Sirweb expert system (i2a, Pérols Cedex, France), operated in our unit. The *V. harveyi* isolate could not be tested since it did not grow sufficiently at 35°C on mueller hinton agar. 

## 6. Final Outcome

**Figure 2:**
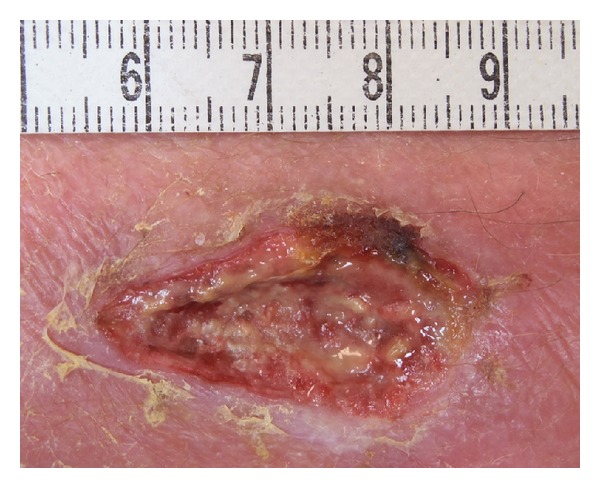
Wound, following seven days of ofloxacin (Tarivid) and following daily debridement, eight days after opening by removing the surgical threat. (Bar at the top is in cm.)

The wound improved well under empiric ofloxacin therapy and daily care (debridement) (Figure 2). Following report of microbiological results, the antibiotic regime was changed to 100 mg doxycycline twice daily (one of the antimicrobials recommended in the treatment of *Vibrio vulnificus* infections [12]) for another 10 days. Surgical wound debridement continued overall for three weeks. The wound healed without further complications though it took 14 weeks until complete closure of the wound was noticed.

## 7. Discussion

The subspecies *piscicida* of the bacterium *P. damselae* is a very well-studied fish pathogen, causing a disease known as “fish pasteurellosis” [23]. Human diseases caused by *P. damselae* subspecies *damselae* (originally described as *Vibrio damsela* [24]) are rare, with less than 20 patients reported in the literature. Like other *Vibrionaceae* species its natural habitat is seawater. The second microorganism reported here, *V. harveyi*, is considered nonpathogenic to humans [25]. However, the bacterium was recovered from the wound more than one week after the initial injury and the presumed contact with this bacterium. Under laboratory conditions, *V. harveyi* shows optimal growth at 20°C [26]. During our routine work, a reduced growth rate at 35°C was observed (susceptibility test could not be read due to sparse growth). It seems therefore not very likely that *V. harveyi* is able to proliferate in human tissue. Therefore, the actual role of this micro-organism in the etiology of the wound infection remains unclear, with a role as colonizing bacterium being equally likely as a role as (secondary) pathogen. While mixed infections with *P. damselae* and *V. parahaemolyticus* or *V. alginolyticus* have been mentioned in the literature [2], the case reported here, presenting as a mixed infection of *P. damselae* with *V. harveyi*, is to the best of our knowledge the first one reported in the literature. Since the patient denied having taken antibiotics following the initial hospital visit it is likely that the isolated microorganisms were those solely accountable for the infection.

A number of case reports of *P. damselae* wound infections have been published [13–22, 27], and the described infections were mostly severe, in the majority of those cases, and even fatal. The clinical presentation was predominantly necrotizing fasciitis, a condition requiring fast medical response, that is, surgery and/or amputation [15].  Table 3 summarizes relevant information on severe wound infections caused by *P. damselae* as extracted from the available literature [13–22]. Taking this into account, the patient described in our case report showed a very favorable outcome.

Beside infections as a consequence of lacerations or other minor injuries, infections following oral ingestion provide the second way of developing this zoonosis [28]. 

It should be noted, however, that in the literature prior to 2004 the older synonyms were used, making literature search cumbersome. Actually, the species names *Photobacterium damselae*, *Photobacterium damsela*, *Vibrio damsela*, *Vibrio damselae*, *Pasteurella damselae* (for the fish disease causing subspecies *piscicida*), and even *Listonella damselae* had to be used as search terms to retrieve the published cases. 

Human cases might be underreported, given that infections may not be fatal [2]. Prior to the advent of MALDI-TOF in routine laboratories, the identification of *Vibrionaceae* was hampered by the fact that commercial identification systems showed poor reliability in the identification of members of the genus *Vibrio* [29]. In our case, the identification of both bacteria was provided conveniently by MALDI-TOF technique. However, Vitek 2 compact, utilizing an identification card for Gram-negatives (GN-card; bioMérieux, Marcy l'Etoile, France), identified *P. damselae* with a very good score as well. 

Different from other potentially colonizing bacteria, frequently found in wound swabs, *P. damselae* impresses by its massive *β* hemolysis, which usually would alert the microbiologist to start closer examination of such colonies. Therefore it is not likely that wound infections by this pathogen go by undetected. The hemolytic phospholipase *Dly* or damselysin has been identified as one of the main virulence mechanisms. However, a correlation of virulence with the strength of hemolysis has been reported. Only strong hemolytic strains are carrying a plasmid, pPHDD1, which encodes for further virulence determinants, necessary to deploy full virulence [30]. Using primers F-P.dam-3 and R-P.dam-5, the expected amplicon of dly was amplified. Thereby it was demonstrated that the isolated *P. damselae* subspecies *damselae* was positive for plasmid pPHDD1, carrying *dly*, thus being of the highly virulent type, as was already supposed from the massive hemolysis (Figure 1(d)). It is therefore likely that infections with this bacterium, even with the highly virulent phenotype, do not always progress into the most severe form of necrotizing fasciitis and that other factors, probably individual predisposing factors of the patients, contribute to the outcome of each individual case [3]. Most of the patients with necrotizing fasciitis listed in Table 3 were aged around 60 years or above, and some showed underlying tentatively predisposing conditions like diabetes or alcohol abuse. Interestingly, all were males. 

## 8. Conclusion

Our case presentation shows that unusual infections must be suspected in patients returning from abroad and reporting injuries in the countries they have visited. Global travelling nowadays allows taking a bath in the Pacific Ocean and being back at work in Europe not even two days later, providing challenges not only for specialized institutes like departments for tropical medicine but also for office practitioners. Even bacteria supposed to be nonpathogenic, which have marine seawaters as primary habitat, may actually be able to cause disease or at least to participate in infections as has been reported here with the detection of *V. harveyi*. While the etiology reported here is uncommon, severe or fatal infections caused by other vibrionic bacteria are well known. Both *V. parahaemolyticus* and *V. vulnificus* and, to a lesser extent, *V. alginolyticus* infections have been reported, and it has been discussed that global climate change—among other factors—may lead to an increase in infections due to marine bacteria [4, 31]. In view of the high fatality rates observed with wound infections after progressing to necrotizing fasciitis, early and aggressive antimicrobial therapy is essential if *Vibrionaceae* are suspected. Death may occur as early as 20 hrs after onset of initial symptoms [13]. Bacteriology from adequate specimens is imperative to allow for optional adjustment of therapy following the laboratories report. In the most severe cases, even amputation at a very early stage of disease might be required [15, 19].

Modern trends and developments in diagnostics (e.g., MALDI-TOF) allow rapid identification of even unusual pathogens, although frequent taxonomic changes as is the case with *P. damselae* may actually complicate data retrieval. With the wide availability of such techniques it is likely that more infections like the one reported here are detected in the near future. With respect to this, the current case report may add some important information for clinicians as well. Medical microbiologists should always be alerted if MALDI-TOF analysis yields remarkable identifications. 

## Figures and Tables

**Table 1:** MALDI-TOF identification results obtained for *P. damselae* and *V. harveyi*. Note that the number of available spectra in the database is 4 for *P. damselae* (two for each subspecies) and 4 for *V. harveyi*. MALDI scores equal or higher than 2.000 allow for the designation of a species while below 2.000 but equal or above 1.700 allow for the designation of a genus. By default, 10 hits per identification are provided. Hits with scores in support of the final identification are given in bold. The installed database was V3.3.1.0_4110-4613 as of August 2012.

Matching spectra:	Score
***Photobacterium damselae* ssp. *piscicida* PC846_1 EGS**	**2.176**
***Photobacterium damselae* ssp. *piscicida* ATCC 29690 EGS**	**2.155**
***Photobacterium damselae* ssp. *damselae* DSM 7482T HAM**	**2.151**
*Photobacterium damselae* ssp. *damselae* CDC 2227_81 EGS	1.786
*Enterobacter cloacae* 20105_2 CHB	1.427
*Pseudomonas fluorescens* DSM 50090T HAM	1.358
*Klebsiella pneumoniae* ssp. *ozaenae* CCM 5792T CCM	1.328
*Kocuria rosea* IMET 11363T HKJ	1.289
*Comamonas nitrativorans* DSM 13191T HAM	1.279
*Leuconostoc mesenteroides* ssp. *cremoris* DSM 20200 DSM	1.275
***Vibrio harveyi* DSM 19623T DSM**	**2.210**
*Vibrio alginolyticus* CCM 7037 CCM	1.935
*Vibrio parahaemolyticus* DSM 15477 DSM	1.856
*Vibrio harveyi* LMG 19643T HAM	1.850
*Vibrio mytili* DSM 19137T LGL	1.840
*Vibrio parahaemolyticus* DSM 10027T DSM	1.825
*Vibrio parahaemolyticus* DSM 15416 DSM	1.818
*Vibrio parahaemolyticus* DSM 11058 DSM	1.744
*Vibrio alginolyticus* DSM 2171T DSM	1.732
*Vibrio alginolyticus* CCM 2578T CCM	1.727

**Table 2:** Result of disc diffusion test (except amikacin, which was tested with *E*-Test) with *P. damselae* on Mu eller-Hinton agar after 24 hrs of incubation at 35 ± 1°C. The antimicrobials tested are those routinely used for Gram-negative isolates in the laboratory of one of us. The categorical interpretations (S: susceptible; R: resistant) are based on the CLSI recommendation for *Vibrio *ssp. (*excluding Vibrio cholerae*) as implemented in the Sirweb software (i2a, Pérols Cedex, France). Antimicrobials capitalized with “*” are those recommended for first-line testing, those in Ca pitals indicate antimicrobials recommended for testing, and those in *italics* are antibiotics tested routinely in our lab with Gram-negatives on Mueller Hinton agar, but are not recommended by Clinical and Laboratory Standard Institute (CLSI) when testing *Vibrio species*. (n.a.: not applicable).

Antimicrobial	Disc concentration (*μ*g)	Inhibition zone diameter (mm)		Categorical interpretation based on zone
		Observed	Zone diameter breakpoint	
Ampicillin	10	24	≥17	S
Ampicillin/Clavulanic acid	20/10	25	≥15	S
Ampicillin/Sulbactam	10/10	24	≥18	S
Piperacillin/tazobactam	100/10	30	≥21	S
*Cefaclor *	30	31	n.a.	n.a.
Cefotaxime	30	29	≥23	S
Cefoxitin	30	24	≥18	S
*Cefpodoxime *	10	26	n.a.	n.a.
Ceftazidime*	30	30	≥18	S
Imipenem	10	26	≥16	S
Meropenem	10	13	≥16	R
Ciprofloxacin*	5	30	≥21	S
Levofloxacin*	5	28	≥17	S
Amikacin	*E*-test	2 mg/L	≤16 mg/L	S
Gentamicin	10	17	≥15	S
Sulfamethoxazole	1.25/23.75	22	≥16	S
Tetracy c line*	30	28	≥19	S
*Nitrofurantoin *	300	24	n.a.	n.a.

**Table 3:** Review of the available human cases of severe wound infections*, caused by *P. damselae* (for other synonyms found in the literature refer to the discussion section).

Patient	Age	Type of injury, other conditions	Clinical course	Outcome	Reference
Male	58	No obvious injury reported, swollen hand after fishing, and diabetes mellitus	Postsurgical complications, cardiac arrest	Fatal	[13, 14]
Male	76	Minor puncture at thumb	Wound, progressing to necrotizing fasciitis (NF), intravascular coagulation, septic shock	Fatal	[13]
Male	69	Partially healed laceration (finger) and fishhook injury (finger) 2 weeks prior to onset	NF, multiple organ failure	Fatal	[15]
Male	43	Laceration by stingray when stepping off a boat	Fever, erythema, followed by NF	Cured (deep surgical debridement)	[16]
Male	64	Injury from fish hook, atherosclerotic heart disease, and ventricular arrhythmias	Erythema and profound edema, postsurgical complications, acute renal failure	Fatal	[17]
Male	63	No injury recorded, consumption of raw eel, diabetes mellitus, alcoholic, liver disease	Swelling and erythema on arm and hand, NF, intravascular hemolysis, septic shock	Fatal	[18]
Male	70	Knife cut after handling bluefish, previously healthy, mitral valve replacement and coronary artery bypass	Wound infection, fever and swelling of the hand, sepsis	Fatal	[19]
Male	62	Puncture wound at thenar following rabbitfish fin injury	Swollen forearm with dusky discoloration, progressing into NF, renal failure, septic shock	Fatal	[20]
Male	38	Fish fin puncture	erythema and edema, severe pain	Cured by amputation	[21]
Male	61	Minor injury while cleaning catfish, alcoholic, and diabetes mellitus	NF, intravasal coagulation, renal failure, cardiac arrest	Fatal	[22]
Male	64	Laceration injury in marine environment	Wound infection with local necrosis	Cured	This report

*Another six cases of *P. damselae* wound infections are given in [2] without enough information to be included in this table.
